# Gut-derived fungemia due to *Kodamaea ohmeri* combined with invasive pulmonary aspergillosis: a case report

**DOI:** 10.1186/s12879-022-07866-6

**Published:** 2022-12-03

**Authors:** Zi-Mu Li, Yu-Kun Kuang, Yi-Fan Zheng, Pei-Hang Xu, Ji-Yu Wang, Run-Jing Gan, Hui-Xia Li, Li-Hong Bai, Can-Mao Xie, Ke-Jing Tang

**Affiliations:** 1 Division of Pulmonary and Critical Care Medicine, The First Affiliated Hospital, Sun Yat-Sen University, No. 58, Zhongshan Er Lu, Guangzhou, 510080 China; 2 Department of Pharmacy, The First Affiliated Hospital, Sun Yat-Sen University, Guangzhou, China; 3 Institute of Pulmonary Diseases, Sun Yat-Sen University, Guangzhou, China

**Keywords:** *Kodamaea ohmeri*, Gut-derived fungemia, Invasive pulmonary aspergillosis, Case report

## Abstract

**Background:**

*Kodamaea ohmeri* is a rare pathogen with high mortality and is found among blood samples in a considerable proportion; however, gastrointestinal infection of *K. ohmeri* is extremely rare. Invasive pulmonary aspergillosis is also an uncommon fungal; these two fungal infections reported concomitantly are unprecedented.

**Case presentation:**

We described a case of a 37-year-old male who got infected with *K. ohmeri* and invasive pulmonary aspergillosis. We used the mass spectrometry and histopathology to identify these two fungal infections separately. For the treatment of *K. ohmeri*, we chose caspofungin. As for invasive pulmonary aspergillosis, we used voriconazole, amphotericin B, and then surgery. The patient was treated successfully through the collaboration of multiple disciplines.

**Conclusions:**

We speculate that the destruction of the intestinal mucosa barrier can make the intestine one of the ways for certain fungi to infect the human body.

## Background

*Kodamaea ohmeri* belongs to the class Ascomycetes and the family Saccharomycetaceae [1]. *K. ohmeri* is commonly used in the food industry, especially in the preparation of pickles because of its fermentation capability. [1] It is a rare pathogen and is reported to cause high mortality (50%) in pediatric populations [1]. The pathogen, firstly isolated clinically in 1984 from pleural fluid, was initially considered a contaminant. [2]. The first case of fungemia caused by *K. ohmeri* was described in 1998, when a 48-year-old patient with diabetes who had been taking immunosuppressive drugs for a long time died of it [2]. Since then, the pathogen has gradually entered the vision of clinicians. Fever and chills were the most common clinical presentations in patients who developed invasive infections of *K. ohmeri* [3, 4]. Respiratory distress and disturbance of consciousness were reported as the patient progressed to sepsis in fungemia cases [3]. Besides, different infection types had different local symptoms, for example, abdominal pain and hematuria often occurred in patients who had peritonitis and urinary tract infection. Local redness, swelling, and pain often occurred if the infection site was explicit (such as the catheter insertion site and wound infection) [3].

Invasive pulmonary aspergillosis (IPA) infection is mostly caused by the inhalation of Aspergillus spores, which is a ubiquitous and saprophytic filamentous fungus. It is a serious complication with high mortality and poor prognosis, so the earlier detection and initiation of treatment are needed [5]. However, the definitive diagnosis is often difficult to make in clinical settings and it causes unacceptably high mortality despite standard of care treatment [6].

In this report, we described a case of *K. ohmeri* fungemia derived from the gut with invasive pulmonary aspergillosis in a young man who was treated successfully with the collective efforts from multiple departments including the Gastroenterology Department, the Division of Pulmonary and Critical Care Medicine, and the Department of Thoracic Surgery.

## Case presentation

**Fig. 1:**
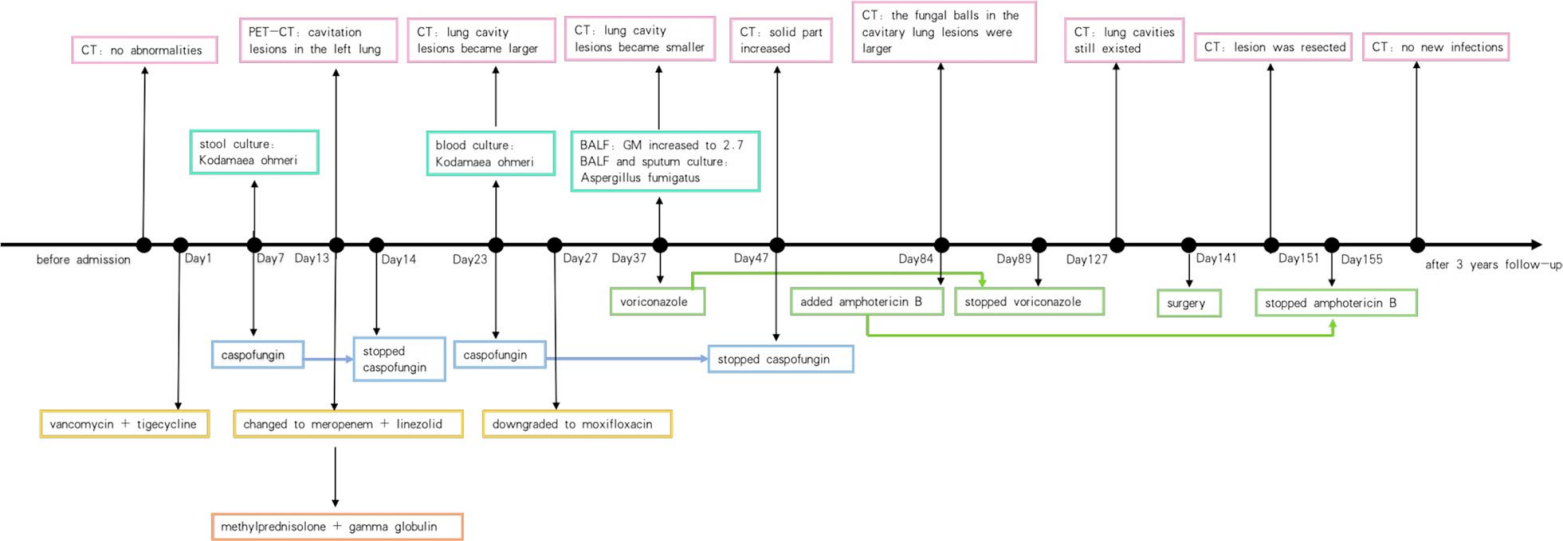
The course of diagnosis and treatment of the case

This case report is based on a 37-year-old male living with his wife in Guangdong, China. He denied any history of immunodeficiency disease and relevant medication and intravenous drug addiction (Fig. 1).

**Fig. 2:**
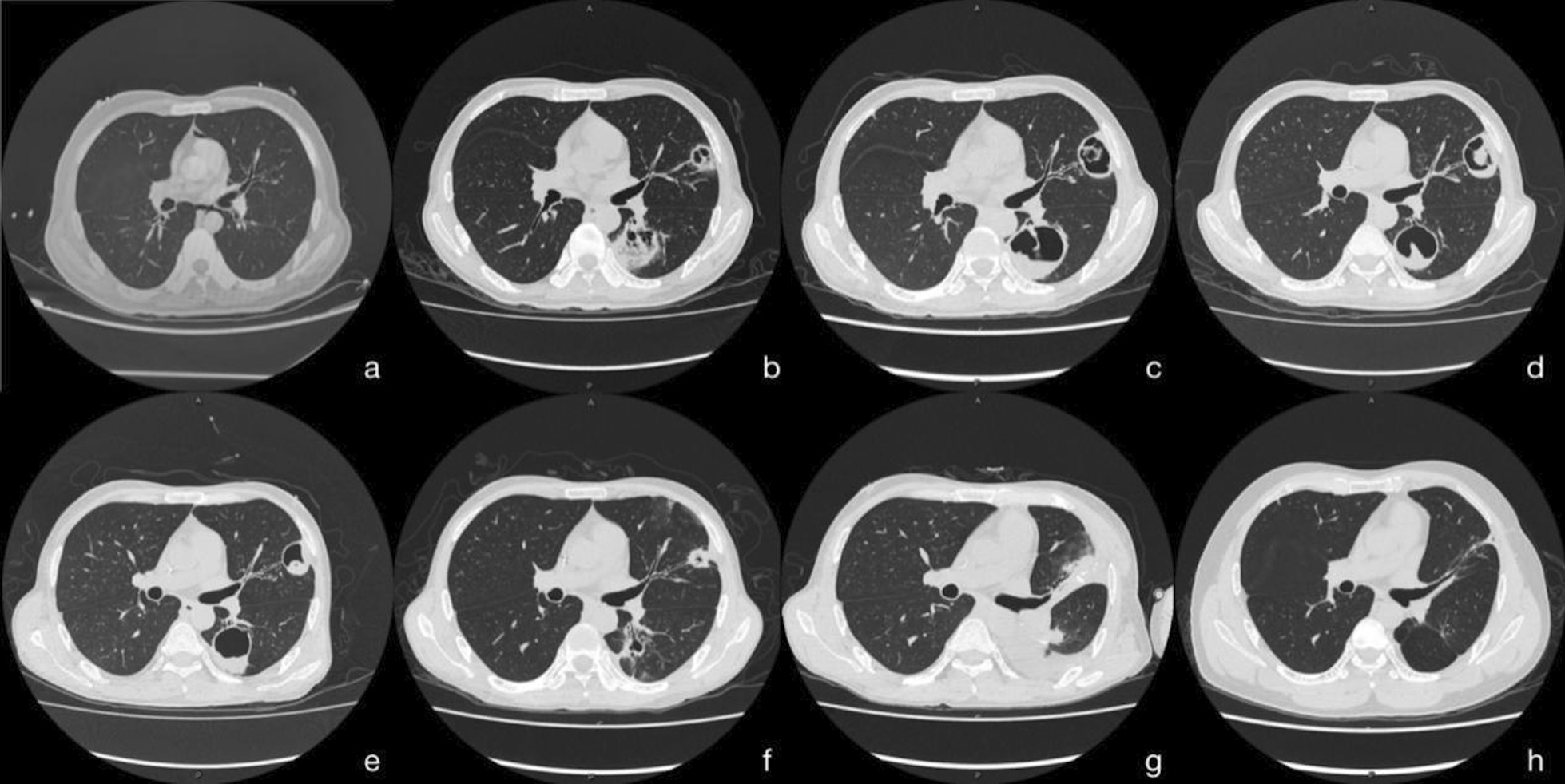
**a** Chest CT showed no abnormalities before admission to our hospital. **b** PET-CT showed cavitary lung lesions on day 13 after admission. **c** Chest CT showed that the cavitary lung lesions were slightly larger than before on day 23 after admission. **d** chest CT revealed increased consolidation in the cavitary lung lesions on day 37 after admission. **e** chest CT revealed that the cavitary lung lesions were smaller than before on day 47 after admission. **f** chest CT showed that the lung cavities still existed, and the exudates around the lesion got slightly reduced on day 127 after admission. **g** chest CT showed that the upper lingual and dorsal left lower lobe lesions of the left upper lobe were removed 10 days after operation (on day 151 after admission). **h** chest CT showed no new infections after 3 years follow-up

Initially, the patient developed a fever with a peak temperature of 40 °C after drinking Chinese herbal wine (the specific ingredients were unknown), accompanied with chills, shivers, rash, and pruritus. After a week, abdominal colic gradually appeared, with watery diarrhea 20–30 times a day. The patient visited several hospitals. The highest white blood cell count was 51.70 × 10^9^/L (reference value 4.00–10.00 × 10^9^/L) and the neutrophil ratio was 87.0% (reference value 46–75%). The chest CT showed no abnormalities (Fig. 2a) and the abdominal CT showed diffuse bowel wall edema. With a variety of broad-spectrum antibiotics and supportive treatment in the local hospital for 40 days, his general condition did not improve, and he was transferred to the emergency department of our hospital afterward.

**Fig. 3:**
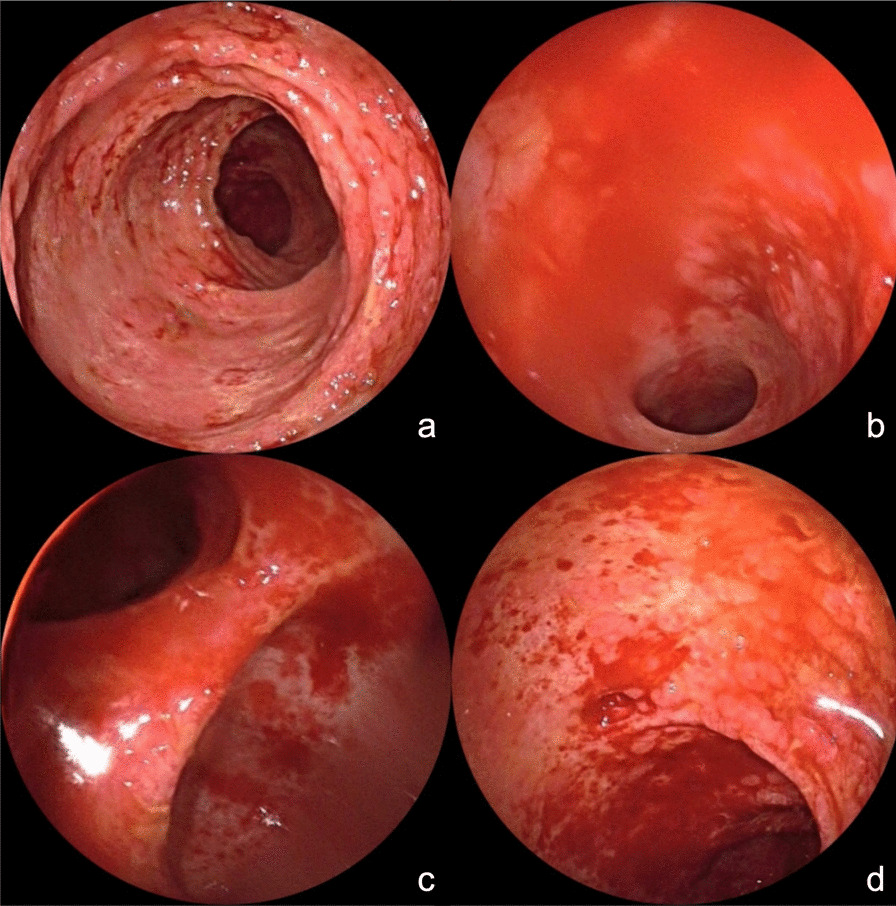
**a–d** Colonoscopy showed diffuse congestion, edema, erosion, and spontaneous blood in the colonic mucosa, and the colonic band structure disappeared

During the treatment in the emergency department of our hospital, the patient had a fever of 39 °C, with hypotension of 89/54 mmHg, malnutrition, skin flushing, scattered flaky rash and exfoliation, tenderness and rebound pain in the lower abdomen. Laboratory tests showed a white blood cell count of 3.84 × 109/L, neutrophil ratio of 75.8%, red blood cell count of 3.16 × 1012/L (reference value 4.00–5.50 × 1012/L), hemoglobin of 66 g/L (reference value 130–175 g/L), platelet count of 41 × 109/L (reference value 100–300 × 109/L), C-reactive protein of 24 mg/L (reference value 0–10 mg/L), procalcitonin of 11 ng/ml (reference value 0.00–0.05 ng/ml). The abdominal CT showed an extensive bowel wall edema in the jejunum, ileum, and colon. The colonoscopy showed a diffuse congestion, edema, erosion, and spontaneous blood in the colonic mucosa, and the colonic band structure disappeared (Fig. 3). After admission, multiple blood culture results were negative. The vancomycin and tigecycline were given empirically accompanied by nutritional support treatment. On day 7 after admission, the patient's stool culture test result turned out to be *K. ohmeri* (28 °C, Sabouraud medium), so caspofungin was added to the treatment (for 7 days). He was transferred to the Gastroenterology Department for further treatment on day 9 after admission.

**Fig. 4:**
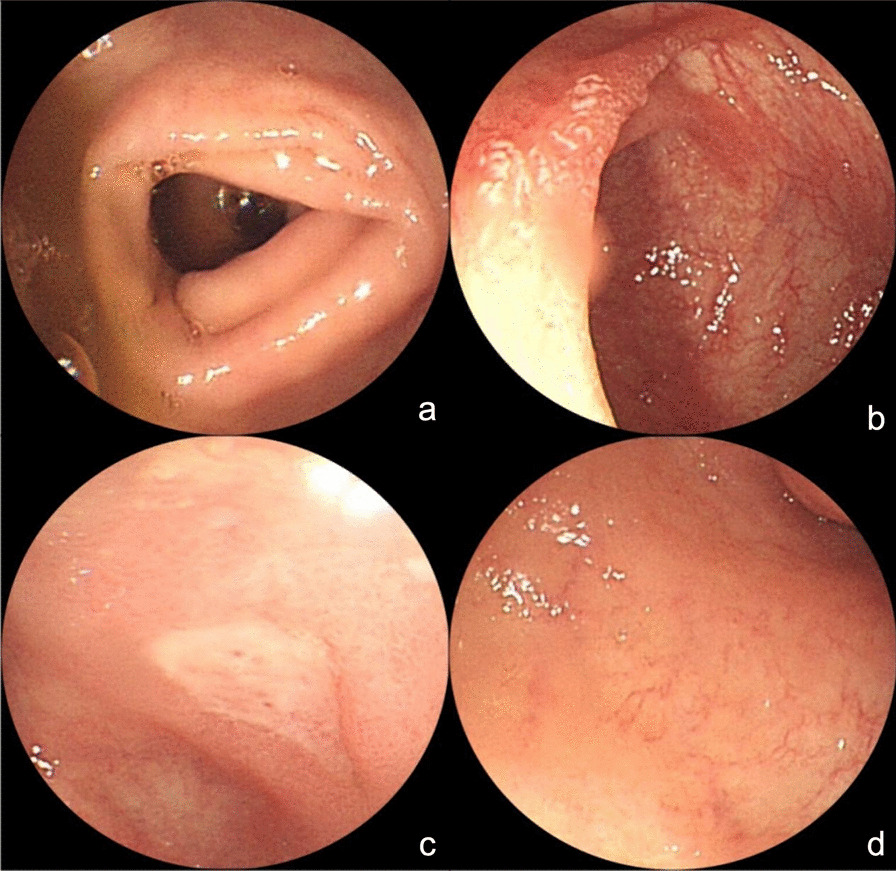
**a–d** Colonoscopy after the treatment with methylprednisolone and gamma globulin revealed that the bowel wall edema was significantly reduced

After the patient was transferred to the Department of Gastroenterology, he still had a fever of 37.3–39.0 °C. The rash, extirpative dermatitis, diarrhea, and abdominal pain were not significantly improved. The WBC count was 16.53 × 109/L, neutrophil ratio of 80.2%, C-reactive protein was 22.9 mg/L, procalcitonin was 0.24 ng/ mL, and blood IgE was 351.30 IU/ mL (reference value was 0.00–120.00 IU/ mL). On day 13 after admission, the PET-CT showed hepatomegaly, splenomegaly. The metabolism was active (SUVmax of the liver was 5.1, SUVmax of the spleen was 5.3); the metabolism of axial bone and proximal long bones of the outer limbs were increased (SUVmax 10.0); cavity lesions in the left lung (Fig. 2b), and metabolism was active (SUVmax was 5.3); the bowel wall of the jejunum, ileum, colon, and rectum had an extensive edema, and the metabolism was slightly increased (SUVmax is 5.0). After a multidisciplinary consultation, considering the poor control of the patient’s fever and leukocytosis, the anti-infective regimen was switched to meropenem combined with linezolid. Considering the erythroderma as well as allergic enteritis may be caused by the Chinese herbal medicine wine, the intravenous methylprednisolone (0.8 mg/Kg/day) was started, supplemented with high-dose intravenous gamma globulin (0.4 mg/Kg/day, for 5 days).The montmorillonite powder was given to relieve diarrhea, drotaverine hydrochloride to relieve pain, and other treatment to correct water and electrolyte disorders and supply nutrition. After the above comprehensive treatment, the peak temperature of the patient was lower than before, fluctuating between 36.5 and 38.0 °C. The abdominal pain was slightly relieved and watery stools decreased to about 10 times a day. A re-examination of abdominal CT revealed that the bowel wall edema was significantly reduced compared to before, and the follow-up re-examination of colonoscopy also confirmed the CT results (Fig. 4).

**Fig. 5:**
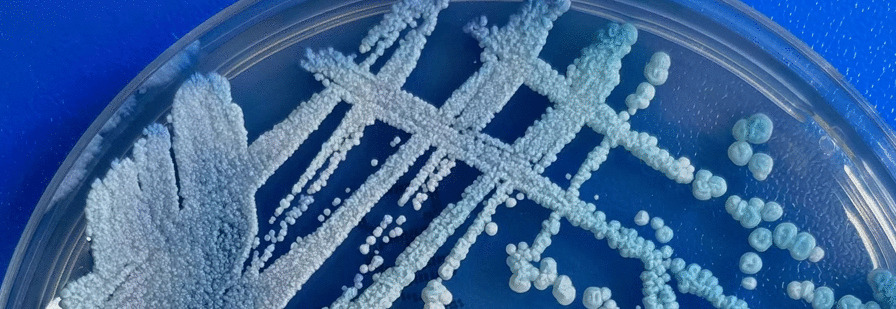
the change in colony color from pink to blue within 48 h on CHROMagar Candida media indicated yeast-like fungal growth

On day 23 (10 days of meropenem combined with linezolid treatment), the patient's body temperature suddenly increased to 39.5 °C with chills. Re-examination of the chest CT showed that the cavitary lung lesions were slightly larger than before (Fig. 2c), so bronchoalveolar lavage was recommended for further detection of pulmonary pathogens. The blood culture showed yeast-like fungal growth (28 °C, CHROMagar Candida media) (Fig. 5), and the MALDI TOF–MS (VITEK MS, bioMérieux, France) showed K. ohmeri. As a result, caspofungin was added to the treatment again. After 24 h’ treatment of caspofungin, the patient defervesced and the blood culture did not change into negative until 9 days after the treatment. On day 27 (after 2 weeks of combined treatment of meropenem and linezolid) The anti-infective therapy was changed to intravenous moxifloxacin. Bronchoscopy was performed and the galactomannan test of bronchoalveolar lavage fluid (BALF) was 1.1 (reference value 0.0–0.9). To further clarify the nature of the lung disease, the patient was transferred to the Devision of Pulmonary and Critical Care Medicine. On day 37 after admission, a cough and sputum appeared gradually. The re-examination of chest CT revealed increased consolidation in the cavitary lung lesions (Fig. 2d). Bronchoscopy was conducted again and the galactomannan test of BALF was 2.7, which was higher than before, and the sputum and BALF culture showed the growth of aspergillus fumigatus, so the clinical diagnosis was invasive pulmonary aspergillosis. Based on the use of caspofungin for the treatment of K. ohmeri, intravenous voriconazole was administrated for anti-aspergillus treatment. On day 47 after admission (10 days after treatment of voriconazole), the re-examination of chest CT revealed that the cavitary lung lesions were smaller than before (Fig. 2e). The general condition of the patient improved, with no fever, subsided red rash on the head and face, relieved cough and abdominal pain, reduced stool frequency which was about 3–5 times, and no more watery stools but yellow paste-like stools. According to the above, the intravenous voriconazole was changed to oral administration, the caspofungin was stopped (24 days in total), the methylprednisolone was tapered to 12 mg/d, and the patient was discharged on day 51 after admission.

**Fig. 6:**
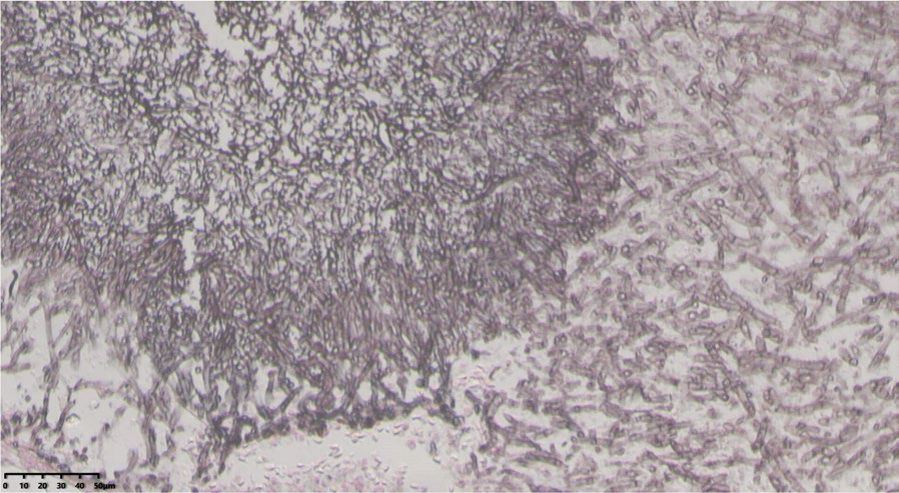
postoperative pathology proved to be invasive Pulmonary aspergillosis (Grocott, 40X)

After discharge, the patient continued oral voriconazole, and the dose of methylprednisolone was reduced to 4 mg/d. One month and a few days later (on day 84 after admission), the patient returned to the hospital for re-examination. Compared with the previous chest CT, the fungal balls in the cavitary lung lesions were larger than before. Therefore, based on treatment of voriconazole (already used for 46 days), intravenous amphotericin B was added for anti-aspergillus therapy, the dose of which is gradually increased to the maximum tolerated dose of 45 mg (0.9 mg/Kg). We also injected 5 mg of amphotericin B into the lung cavity twice under bronchoscope. Voriconazole was discontinued (52 days in total) when amphotericin B was used for 6 days, and methylprednisolone was discontinued at the same time. Six weeks after the use of amphotericin B (total dose 2 g) (on day 127 after admission), the re-examination of chest CT showed that the lung cavities still existed, and exudates around the lesion was slightly reduced (Fig. 2f). Considering the poor efficacy of antifungal treatment, the patient was transferred to the Department of Thoracic Surgery of our hospital 2 weeks later (on day 141 after admission) for video-assisted thoracoscopic wedge resection of the left lower lobe and left upper lingual lobe. Pathological findings of the surgically resected specimen further confirmed the diagnosis of invasive pulmonary aspergillosis (Fig. 6). Re-examination of chest CT 10 days after operation (on day 151 after admission) (Fig. 2g) showed that the upper lingual and dorsal left lower lobe lesions of the left upper lobe were resected, with some exudates and consolidation in the operation area. Amphotericin B was continued for 2 weeks after the operation until the total dose was 3 g. The patient has been followed up for 3 years and has no symptoms such as fever, abdominal pain, and diarrhea, and no new infections have been found on chest CT (Fig. 2h).

## Discussion and conclusions

We searched studies before February 2022 in PubMed, Embase, Web of Science, and CNKI databases (China National Knowledge Infrastructure) with the following text-words: (Kodamaea OR Pichia OR Yamadazyma) AND ohmeri. A total of 52 papers were retrieved and a total of 68 patients were reported. [2, 7–57] *Kodamaea ohmeri* fungemia was in the majority with a total of 47 patients (47/68, 74.6%). [2, 7, 10, 11, 16–26, 28, 29, 31, 34, 35, 37, 38, 40–42, 45–49, 51–53, 57] Other less reported *K. ohmeri* infections including endocarditis, peritonitis, urinary tract infection, pneumonia, keratitis, skin and soft tissue infections, onychomycoses, oral disease, disseminated infection, etc. We reported here for the first time that *K. ohmeri* was cultivated in feces from a patient, and we subsequently discovered that the pathogen entered the bloodstream from the intestinal tract and caused *K. ohmeri* fungemia.

From previous case reports, the risk factors for *K. ohmeri* infection in adults include central venous catheter placement, long stay in ICU, broad-spectrum antibiotics, diabetes, malignant tumors, drug abuse, postoperative prosthetic valve replacement, chronic renal function exhaustion, and so on [4, 48]. In literature reports, most of the fungaemia are related to central venous catheters, and some are also associated with skin and soft tissue destruction or infection, which suggests that mucosa barrier destruction may be one of the important risk factors for *K. ohmeri* bloodstream infection. [56] The patient took the homemade Chinese herbal medicinal wine before the onset of the illness. The brewing of wine requires the process of fermentation by wine yeast. Studies have reported that 171 yeasts were isolated from a traditional alcoholic beverage produced in some African countries—raffia wine and *Saccharomyces cerevisiae* was the predominant species, followed by *Kodamaea ohmeri* (20.4%) [58]. Therefore, it was speculated that *K. ohmeri* entered the bloodstream through the damaged intestinal mucosa and the Chinese herbal medicine wine could not be ruled out as the source of fungus infection. Unfortunately, we did not get any samples of the wine, and we did not conduct any tests for the etiology.

In this case, we confirmed the blood infection of *K. ohmeri* by two methods: initial identification was made by the change in colony color from pink to blue within 48 h on CHROMagar Candida media (Fig. 5), and then MALDI TOF–MS was performed to further confirm the finding. According to the high fatality rate of *K. ohmeri* fungemia, timely and appropriate treatment is needed. Removal of primary infective foci, such as central venous catheters or other invasive medical devices, should be followed by prompt antifungal therapy. We ordered caspofungin for the patient twice when the stool culture and blood culture was positive, as a result, the patient's symptoms improved, and *K. ohmeri* was not cultured in blood sample any more. Although recently it has been pointed out that amphotericin B has a higher success rate of treatment, and choosing echinocandins may lead to treatment failure [4]. This case can present a successful treatment reference for future clinical work.

In this case, the patient developed progressively enlarged lung cavity lesions. there are very few (only 2 cases found by BALF culture) reports of *K. ohmeri* infection in the lungs, and none of them described lung imaging as cavity lesions [59], so we must be alert to possible infections caused by other pathogens in the lungs. The diagnosis of invasive pulmonary aspergillosis (IPA) in this case was determined based on BALF culture, GM test and the pathological diagnosis of the surgically resected specimen.[60] For IPA, voriconazole is the treatment of choice [60], but in this case, the outcome of the chest CT after antifungal treatment implied no significant improvement. Considering his general condition and immune status, timely surgery is necessary. [60]

To the best of our knowledge, this is the first report of *K. ohmeri* detected in the stool and cause gut-derived fungemia accompanied with IPA. In summary, we speculate that the destruction of the intestinal mucosa barrier can make the intestine one of the ways for certain fungi to infect the human body.

## Data Availability

All data generated or analyzed during this study are included in this published article.

## Abbreviations

*K. ohmeri*: *Kodamaea ohmeri*
IPA: Invasive pulmonary aspergillosis
